# Targeting the ACE/Ang II Axis with Captopril Attenuates Neuroinflammation and Cerebral Injury Following Fat Embolism

**DOI:** 10.3390/cells15171550

**Published:** 2026-08-27

**Authors:** Shu Cheng Chen, Chin-Kuo Lin, Cheng-Huang Shen, Michael W. Y. Chan, Zheng-Wei Chen, Yu-Hao Lin, Chung-Sheng Shi, Chieh-Mo Lin, Tzu Hsiung Huang, Justin Ching Hsien Lu, Jui-Ming Sun, Kwok-Tung Lu, Yi-Ling Yang

**Affiliations:** 1 Department of Biomedical Sciences, National Chung Cheng University, Min-Hsiung, Chia-Yi 621, Taiwan; shucheng0615@gmail.com (S.C.C.); biowyc@ccu.edu.tw (M.W.Y.C.); 2 Division of Pulmonary Infection and Critical Care, Department of Pulmonary and Critical Care Medicine, Chang Gung Memorial Hospital, Chia-Yi 613, Taiwan; lingh@cgmh.org.tw (C.-K.L.); f124510714@cgmh.org.tw (C.-M.L.); 3 College of Medicine, Chang Gung University, Taoyuan City 333, Taiwan; 4 Department of Urology, Ditmanson Medical Foundation Chiayi Christian Hospital, Chia-Yi 600, Taiwan; 01712@cych.org.tw; 5 Department of Biochemical Science and Technology, National Chia-Yi University, Chia-Yi 600, Taiwan; aaa2731249@gmail.com; 6 Institute of Biochemistry and Molecular Biology, National Yang Ming Chiao Tung University, Taipei 112, Taiwan; douglas4387863@gmail.com; 7 Graduate Institute of Clinical Medicine Sciences, College of Medicine, Chang Gung University, Taoyuan City 333, Taiwan; csshi@mail.cgu.edu.tw; 8 Department of Respiratory Therapy, Chang Gung Memorial Hospital, Chia-Yi 613, Taiwan; world@cgmh.org.tw; 9 Department of Internal Medicine, Henry Ford St. John Hospital, 22101 Moross Rd, Detroit, MI 48236, USA; jlu3@hfhs.org; 10 Section of Neurosurgery, Department of Surgery, Ditmanson Medical Foundation, Chia-Yi Christian Hospital, Chia-Yi 613, Taiwan; 07178@cych.org.tw; 11 Department of Nursing, School of Nursing, Fooyin University, Kaohsiung 831, Taiwan; 12 Department of Life Science, National Taiwan Normal University, Taipei 106, Taiwan

**Keywords:** fat embolism, RAS, CFE, ACE, captopril, neuroinflammation, neuronal damage

## Abstract

**Background:** Fat embolism (FE) is a severe complication following long-bone fractures. While the initial mechanical insult primarily affects the pulmonary vasculature, the subsequent systemic hypoxia and inflammatory cascades frequently lead to cerebral fat embolism (CFE). This progression highlights a critical, yet under-explored, pathogenic crosstalk within the lung-brain axis. The Renin–Angiotensin System (RAS), particularly the Angiotensin-Converting Enzyme (ACE)/Angiotensin II (Ang II) pathway, is a potent driver of inflammation and is highly expressed in both pulmonary and cerebral tissues. This study aimed to investigate whether modulating this axis with captopril, a specific ACE inhibitor, could attenuate the lung-brain inflammatory crosstalk and protect against CFE-induced neuroinflammation. **Methods:** FE was induced in rats via intravenous injection of fat micelles. Captopril (1, 5, 10, or 20 mg/kg) was administered immediately post-induction. Brain injury was assessed using Oil Red O and H&E staining, brain water content, and malondialdehyde (MDA) assays. ACE expression and localization were evaluated via Western blot and immunofluorescence. The RAS balance was assessed by quantifying Ang II and Ang-(1–7) levels using ELISA. Inflammatory and hypoxic markers, including iNOS, HIF-1α, and phosphorylated ERK (p-ERK), were analyzed by Western blot. **Results:** In this study, we observed rapid microvascular occlusion, brain edema, and neuronal damage within 2 h. FE triggered a significant upregulation of ACE, predominantly localized in cortical astrocytes, alongside elevated oxidative stress (MDA) and Ang II levels. Systemic administration of captopril (5 mg/kg) significantly mitigated these cerebral injuries. Captopril not only suppressed local astrocytic ACE expression and Ang II release but also restored the neuroprotective Angiotensin-(1–7) levels. Furthermore, captopril effectively blunted the hypoxia-driven inflammatory cascades, significantly downregulating iNOS expression and the HIF-1α/VEGF/ERK signaling pathway. **Conclusions:** Our findings suggest that CFE is a consequence of dysregulated lung-brain axis communication, driven by hypoxia and RAS overactivation. By inhibiting ACE, captopril effectively modulates this axis, reducing oxidative stress and neuroinflammation. Targeting the ACE/Ang II pathway represents a promising therapeutic strategy for managing the neurological complications of FE syndrome.

## 1. Introduction

Fat embolism (FE) occurs in most patients following long-bone fractures. When these emboli cause organ dysfunction—primarily respiratory, neurological, and dermatological—the condition is termed fat embolism syndrome (FES). FES also occurs in pelvic trauma, bone marrow transplantation, liposuction, osteomyelitis, or pancreatitis, carrying a mortality rate of approximately 20% [1,2]. Cerebral fat embolism (CFE) is a rare but fatal complication where fat emboli enter the systemic circulation and precipitate severe neurological deficits [3]. Symptoms typically manifest 12 to 72 h post-injury, ranging from altered mental status to seizures and coma [4].

The pathophysiology of FES and CFE is believed to result from synergistic mechanical and biochemical processes. The mechanical theory posits that trauma causes the direct entry of fat globules (7–10 μm in diameter) from bone marrow or adipose tissue into veins and marrow sinusoids, which are richly interconnected with the vascular system [4,5]. These fat globules may lodge in the pulmonary vasculature or bypass the pulmonary filter to reach the systemic arterial circulation, causing CFE [3,6]. In some cases, CFE develops without any preceding respiratory symptoms, either because microemboli pass directly into the cerebral circulation or because the patient has sufficient pulmonary reserve [7]. Alongside this mechanical blockage, trauma triggers a biochemical response characterized by systemic inflammation and metabolic dysregulation. During this phase, lipases degrade triglycerides into glycerol and free fatty acids (FFAs) [8,9]. These circulating FFAs are directly cytotoxic. They damage the endothelium, increase capillary permeability, and promote edema while activating inflammatory cascades, including IL-1 and inflammasome pathways [10]. The presence of fat emboli also activates the coagulation cascade, leading to microthrombi that worsen cerebral ischemia and hypoperfusion [8]. Because no specific pharmacological treatments can reverse this complex cascade, clinical management of CFE relies heavily on supportive care.

The pathogenesis of FES provides a quintessential model of the lung-brain axis in acute trauma. According to the mechanical theory, marrow-derived fat globules first enter the venous circulation and inevitably lodge in the pulmonary capillary bed. This initial pulmonary insult not only causes acute right ventricular strain and respiratory distress but also triggers a massive release of systemic inflammatory mediators and induces profound systemic hypoxia [11,12]. The brain, being highly sensitive to oxygen deprivation and systemic inflammation, becomes the secondary victim. Hypoxia and circulating cytokines compromise the blood–brain barrier (BBB), allowing inflammatory signals—and potentially micro-emboli that bypass the pulmonary filter—to enter the cerebral parenchyma, culminating in CFE [4,13,14]. Therefore, understanding and targeting the mediators that bridge pulmonary injury to cerebral inflammation is crucial for therapeutic intervention.

The Renin–Angiotensin System (RAS), traditionally known for maintaining cardiovascular homeostasis, is heavily implicated in this cross-organ communication. The pulmonary endothelium is the primary site of ACE expression and Ang II generation in the body. During acute lung injury, such as in FE, pulmonary ACE is rapidly upregulated, releasing high levels of Ang II into the systemic circulation, which exacerbates systemic inflammation and oxidative stress [12,15,16,17]. It is also evidenced that brain possesses a local RAS and plays an essential role in cerebral function and pathology [18]. The cerebral RAS comprises two opposing pathways. The pathological axis involves angiotensin-converting enzyme (ACE) and angiotensin II (Ang II), which binds to angiotensin type 1 receptors (AT1R) [19]. AT1R activation causes vasoconstriction, oxidative stress, and robust inflammatory responses [20,21,22]. The counter-regulatory protective axis involves ACE2 and angiotensin-(1–7) [Ang-(1–7)], which activates Mas receptors (MasR) to elicit vasodilation and anti-inflammatory effects [23,24]. Evidence suggests that ACE inhibitors, such as captopril, provide significant neuroprotection [18,25]. Captopril readily crosses the blood–brain barrier (BBB) and reduces reactive oxygen species and pro-inflammatory mediators. Because the biochemical phase of CFE is characterized by intense neuroinflammation and endothelial disruption [26], targeting the RAS with a specific ACE inhibitor like captopril presents a compelling therapeutic opportunity. In this study, we hypothesized that modulating the RAS with captopril, a well-established ACE inhibitor capable of acting both systemically and centrally, could effectively disrupt this pathogenic lung-brain crosstalk.

## 2. Materials and Methods

### 2.1. Fat Embolism Induction

Male Wistar rats weighing 350–370 g were purchased from BioLASCO, Taiwan, Co., Ltd., Taipei, Taiwan and housed individually in a temperature-controlled animal colony at 24 °C with a standard 12 h:12 h light/dark cycle. Fat embolism (FE) was induced by injecting 0.2 mL fatty micelles into the tail vein. These micelles were prepared by extracting animal oil from rat adipose tissue and mixing it with water (1:1), a procedure proven to reliably induce FE [10,26]. The fat droplet of 7–10 μm size may pass through the ling and reaches systemic circulation causing embolization to brain, skin, kidney [27,28].

To investigate the role of ACE in CFE and assess the therapeutic potential of captopril, the specific ACE inhibitor was administered immediately following FE induction. All procedures followed the National Institutes of Health Guide for Care and Use of Laboratory Animals and were approved by the Institutional Animal Care and Use Committee (IACUC) at the National Chia-Yi University.

### 2.2. Evaluation of Neuron Damage

Forty-eight male Wistar rats were randomly divided into sham, FE induced, and FE with captopril groups. Following FE induction and drug administration, rats were sacrificed with an overdose of pentobarbital (100 mg/kg, intraperitoneally) and were then transcardially perfused with 0.9% NaCl followed by 10% neutral formalin. Brains were rapidly removed, embedded in paraffin blocks, and sectioned into 5 μm coronal slices. Sections were stained with hematoxylin and eosin (H&E) for microscopic evaluation.

### 2.3. Water Content Measurement

After deep anesthesia with pentobarbital (100 mg/kg), rats were decapitated. Brains were quickly extracted and weighed to record the wet weight. Tissues were then dried at 120 °C for 24 h to obtain the dry weight. Evaluated in a double-blinded manner, brain water content was calculated as [(wet weight − dry weight)/wet weight] × 100% [26].

### 2.4. Malondialdehyde (MDA) Assay

Cerebral lipid peroxidation was evaluated by measuring malondialdehyde (MDA) level using a modified protocol [10]. Briefly, 100 μL of cortical tissue supernatant was mixed with 200 μL of 10% trichloroacetic acid (TCA) and incubated on ice for 15 min for protein precipitation. The mixture was centrifuged at 2200× *g* for 15 min at 4 °C. Subsequently, 200 μL of the supernatant was reacted with an equal volume of 0.67% thiobarbituric acid (TBA) and heated in boiling water (100 °C) for 10 min. Absorbance was measured at 532 nm (U-1900, Hitachi, Minato City, Tokyo, Japan) against standard curve generated using serially diluted malondialdehyde (Thermo Fisher Scientific Inc., Waltham, MA, USA).

### 2.5. Western Blot Analysis

Cortical tissues were homogenized in lysis buffer containing protease inhibitor cocktail (#78430, Thermo Fisher Scientific Inc., Waltham, MA, USA). Proteins were separated by 10% sodium dodecyl sulfate (SDS) gel electrophoresis and transferred to a polyvinylidene difluoride (PVDF) membranes. Membranes were incubated overnight at 4 °C with primary antibodies, against ACE, HIF-1α, ERK, phosphorylated ERK (p-ERK), Raf, and phosphorylated Raf (p-Raf), followed by HRP-conjugated secondary antibodies. Signals were visualized by enhanced chemiluminescence (ECL) assay (Bio Kit Biotechnology, Inc., Miaoli, Taiwan) and analyzed with a Quantity One digital imaging system (Bio-Rad, Hercules, CA, USA). ACE expression was normalized to α-tubulin, while p-ERK and p-Raf were normalized to total ERK and Raf, respectively [10,26].

### 2.6. Enzyme-Linked Immunosorbent Assay (ELISA)

Following euthanasia, cortical tissues were collected, weighed, and rapidly homogenized in ice-cold buffer. Cortical IL-1, Ang 1–7 and Ang II levels were quantified by specific ELISA kits (ELK-7677 and ELK-1400, ELK Biotechnology, Sugar Land, TX, USA) according to the manufacturer’s instructions [10,26].

### 2.7. Immunocytochemical Staining

Brain sections (20 μm) were prepared using a cryostat. After pre-incubation in PBS containing 10% normal goat serum, sections were double-labeling with mouse anti-ACE (1:1000 dilution; Santa Cruz Biotechnology, Inc., Santa Cruz, CA, USA), and goat anti-GFAP (glial marker) (1:1000 dilution; Santa Cruz Biotechnology, Inc., Santa Cruz, CA, USA). Secondary antibodies were AlexaFluor 555 goat anti-mouse IgG (1:3000 dilution, Invitrogen, Carlsbad, CA. USA), and AlexaFluor 488 donkey anti-goat IgG (1:1000 dilution; Invitrogen, Carlsbad, CA. USA). Images were captured using a Nikon Eclipse 80i fluorescence microscope (Nikon, Shinagawa City, Tokyo, Japan).

### 2.8. Statistical Analysis

All data are presented as mean ± standard error of the mean (SEM). Statistical analyses were performed by the non-parametric Mann–Whitney U test or analysis of variance (ANOVA) with Bonferroni–Dunn post hoc. A *p*-value < 0.05 was considered statistically significant.

## 3. Results

### 3.1. Brain Damage Evaluation After CFE

**Figure 1:**
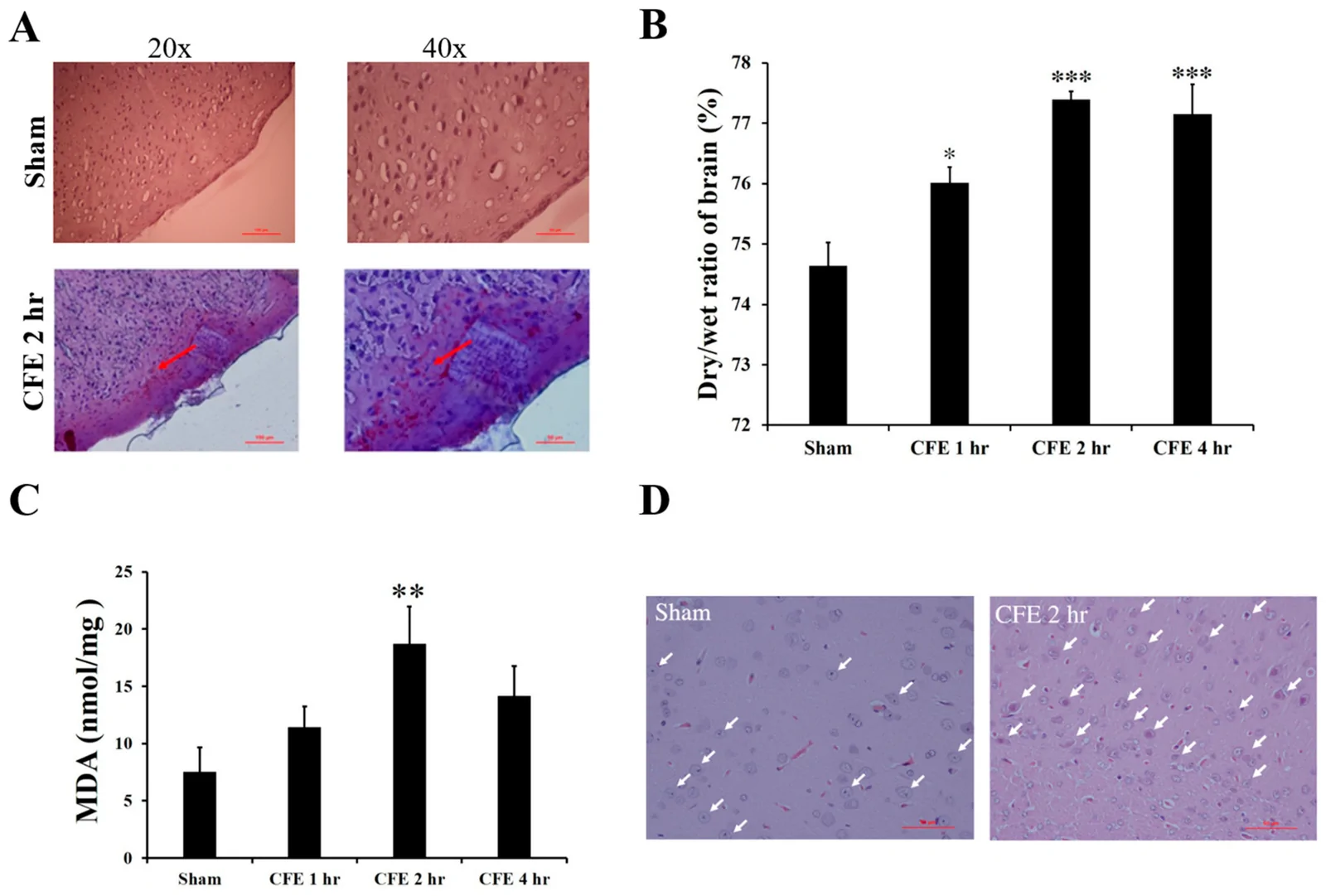
Temporal progression of cerebral injury, edema, and oxidative stress following cerebral fat embolism (CFE). (**A**) Representative images of Oil Red O staining of brain tissues in the Sham and CFE 2 h groups (magnification: 20×, scale bar: 100 μm and magnification: 40×, scale bar: 50 μm). Red arrows indicate the presence of lipid droplets (fat emboli) and subsequent microvascular occlusion. (**B**) Brain water content, evaluated by the dry/wet weight ratio (%), at 1, 2, and 4 h post-CFE. (**C**) Cortical malondialdehyde (MDA) levels at different time points following CFE. (**D**) Representative images of Hematoxylin and Eosin (H&E) staining showing neuronal morphology between sham (Sham) and 2 h post-CFE (CFE 2 h). White arrows indicate damaged, pyknotic, or necrotic neurons. Scale bar: 100 μm. Data are expressed as mean ± SD. * *p* < 0.05, ** *p* < 0.01, *** *p* < 0.001 compared with the Sham group.

To evaluate CFE, we first assessed cortical fat deposition. Compared with the sham group, Oil Red O staining revealed marked lipid accumulation in the cortex at 2 h post-CFE (Figure 1A). Additionally, cerebral water content, an index of brain edema, was significantly elevated (Figure 1B). Cortical MDA levels—a marker of lipid peroxidation—peaked at 2 h post-FE (Figure 1C). Hematoxylin and eosin (H&E) staining demonstrated profound cortical neuronal damage characterized by neuronal swelling, shrinkage, and subsequent cell loss (Figure 1D). These results confirm that CFE rapidly induces acute neuronal injury.

### 3.2. Neuroprotective Effects of Captopril

**Figure 2:**
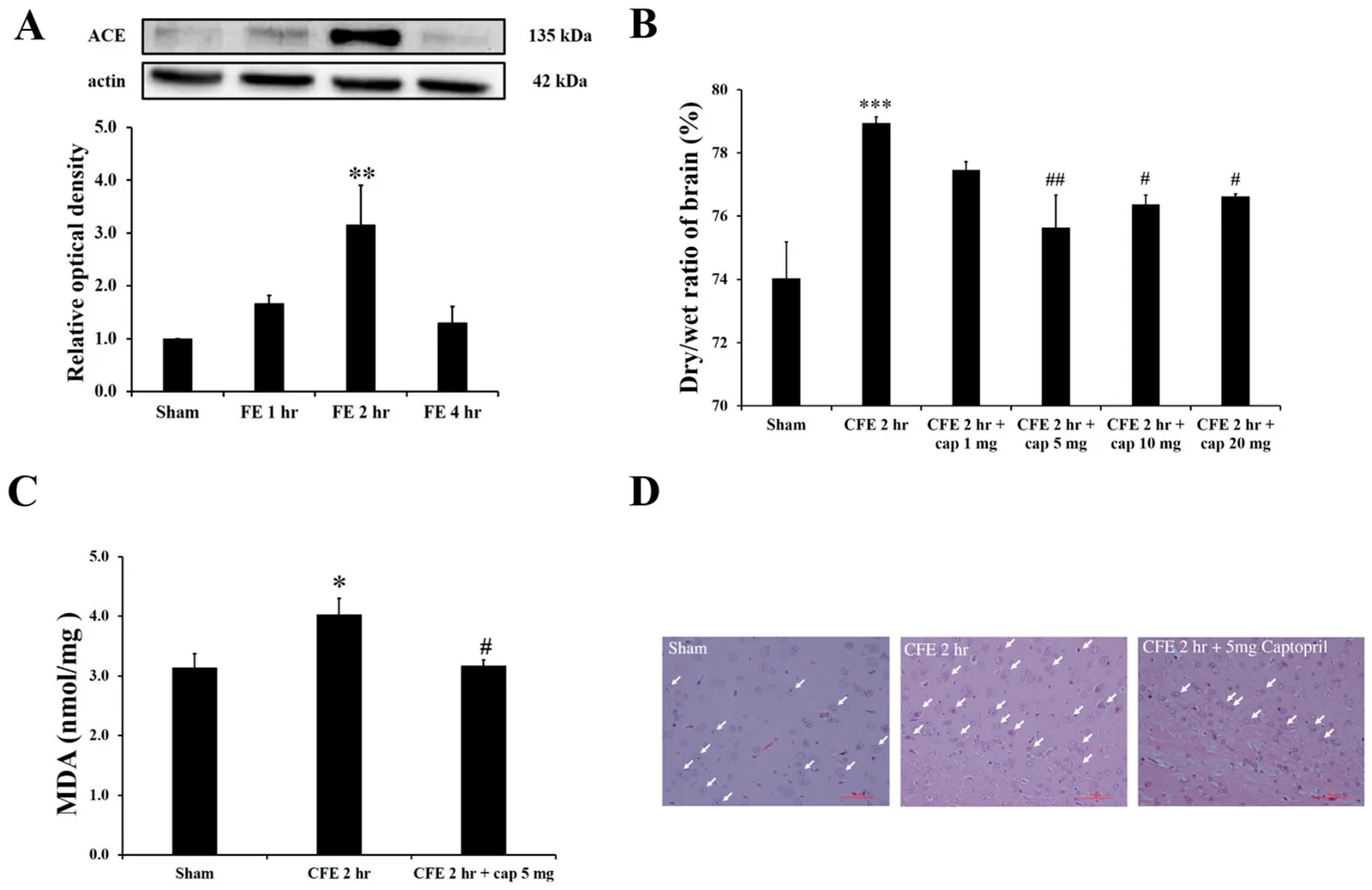
Captopril alleviates CFE-induced brain edema, oxidative stress, and structural damage. (**A**) Western blot analysis and quantitative densitometry of ACE protein expression in the cortex at 1, 2, and 4 h post-CFE. (**B**) Dose-dependent effects of captopril (1, 5, 10, and 20 mg/kg) on brain edema (dry/wet ratio) at 2 h post-CFE. (**C**) Effect of 5 mg/kg captopril treatment on cortical MDA levels at 2 h post-CFE. (**D**) Representative images of hematoxylin and eosin (H&E)-stained brain sections from the sham (Sham), 2 h post-CFE (CFE 2 h), and 2 h post-CFE treated with 5 mg captopril (CFE 2 h + 5 mg Captopril) groups. White arrows indicate damaged or pyknotic neurons. Scale bar: 100 μm. Data are expressed as mean ± SD. * *p* < 0.05, ** *p* < 0.01, *** *p* < 0.001 vs. Sham group; # *p* < 0.05, ## *p* < 0.05 vs. CFE 2 h group.

We next examined the expression dynamics of ACE following FE. Compared to sham controls, ACE protein expression began to rise 1 h post-FE and peaked at 2 h, exhibiting an approximately threefold increase (*p* < 0.05) (Figure 2A). To determine the impact of ACE on CFE, captopril was administered. CFE-induced brain edema was significantly mitigated across multiple captopril doses (1, 5, 10, and 20 mg) (Figure 2B). To minimize potential off-target effects from high dosages, the 5 mg dose was selected for subsequent mechanistic studies. Administration of 5 mg captopril not only significantly reduced MDA levels (Figure 2C) but also markedly attenuated CFE-induced neuronal swelling, shrinkage, and overall morphological damage (Figure 2D).

### 3.3. Mechanisms Underlying the Neuroprotective Effects of Captopril

**Figure 3:**
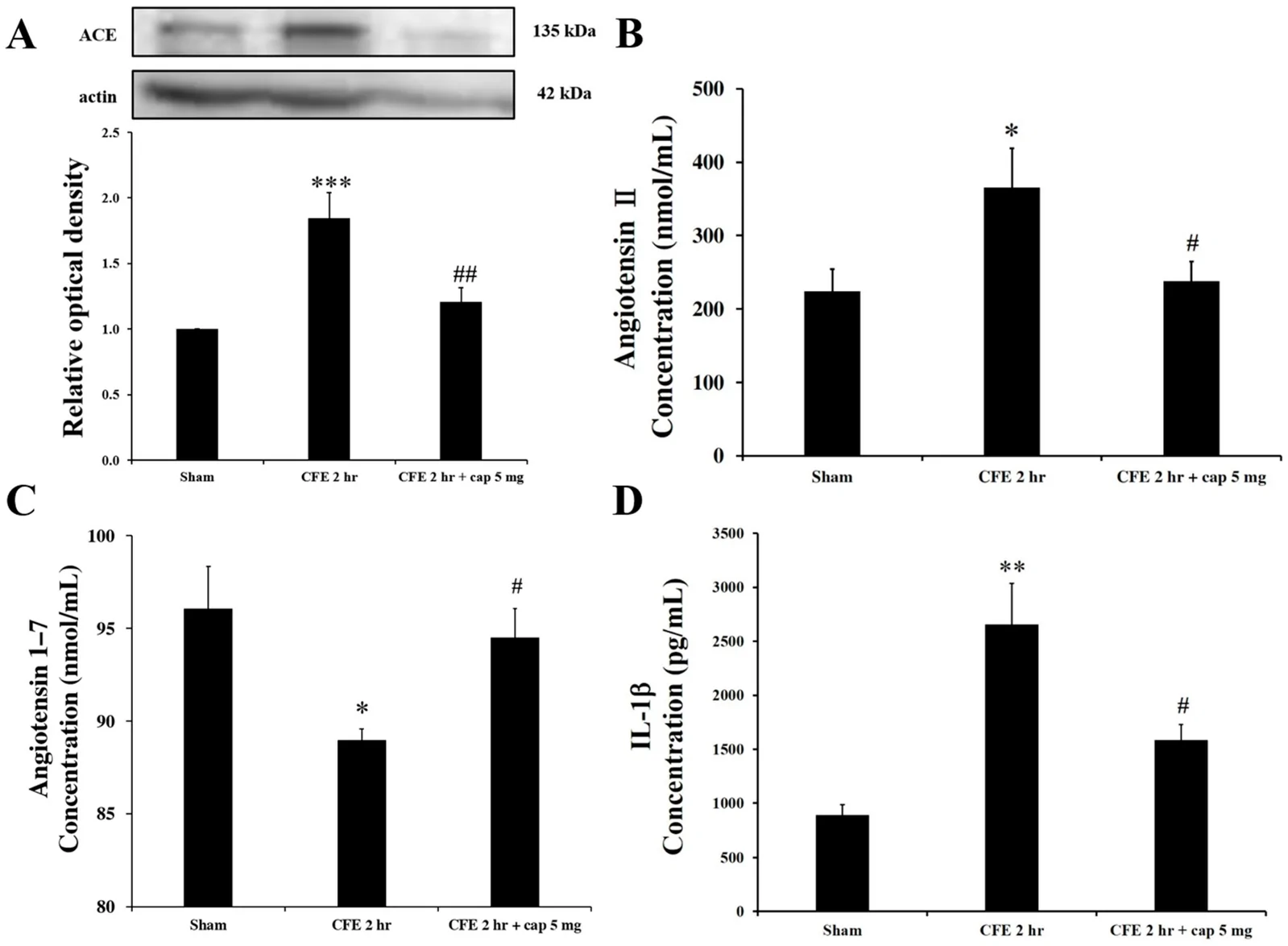
Captopril modulates the cerebral renin-angiotensin system (RAS) following CFE. (**A**) Western blot analysis and relative optical density of ACE protein expression in the Sham, CFE 2 h, and CFE 2 h + captopril (5 mg/kg) groups. (**B**) Concentration of the pro-inflammatory mediator Angiotensin II (nmol/mL) assessed by ELISA. (**C**) Concentration of the neuroprotective peptide Angiotensin 1–7 (nmol/mL). (**D**) Concentration of the IL–1β (nmol/mL). Data are expressed as mean ± SD. * *p* < 0.05, ** *p* < 0.01, *** *p* < 0.001 vs. Sham group; # *p* < 0.05, ## *p* < 0.01 vs. CFE 2 h group.

To elucidate the protective mechanisms of captopril, we assessed its effect on the RAS pathway. Captopril administration antagonized ACE activity and significantly attenuated its protein expression (Figure 3A). Consequently, while FE significantly elevated the levels of the pro-inflammatory mediator Ang II, treatment with 5 mg captopril effectively blunted this release (Figure 3B). In contrast, captopril restored and upregulated the levels of the neuroprotective peptide Ang-(1–7), which had significantly decreased 2 h post-FE (Figure 3C).

**Figure 4:**
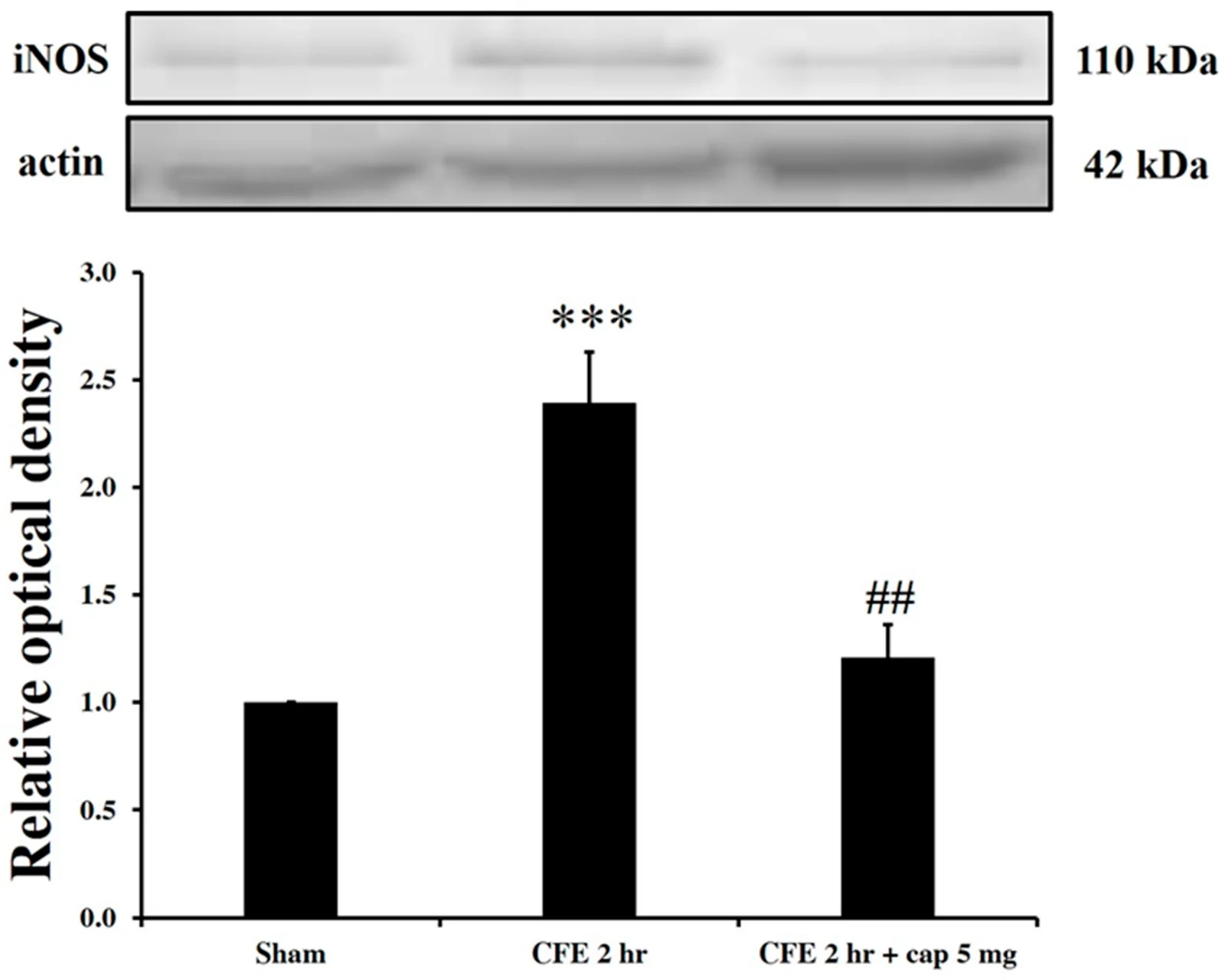
Captopril suppresses CFE-induced iNOS expression. Western blot analysis and quantitative densitometry of inducible nitric oxide synthase (iNOS) protein levels in the cerebral cortex of the Sham, CFE 2 h, and CFE 2 h + captopril (5 mg/kg) groups. β-actin was used as an internal loading control. Data are expressed as mean ± SD. *** *p* < 0.001 vs. Sham group; ## *p* < 0.01 vs. CFE 2 h group.

**Figure 5:**
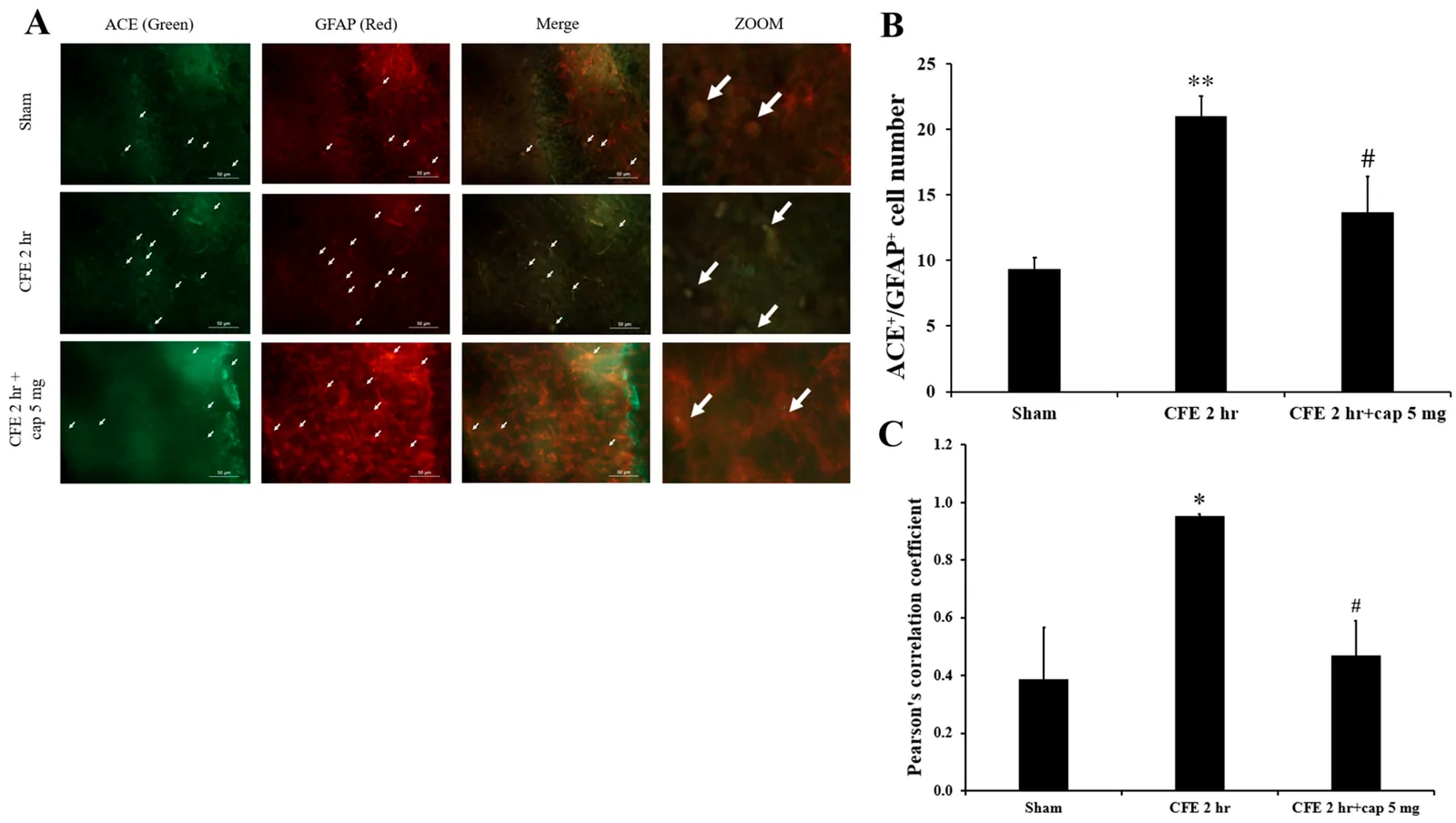
CFE-induced ACE overexpression predominantly colocalizes with astrocytes and is attenuated by captopril. (**A**) Representative immunofluorescence images of the cerebral cortex in the Sham, CFE 2 h, and CFE 2 h + captopril (5 mg/kg) groups. Brain sections were co-stained for ACE (green) and the astrocyte marker GFAP (red). The merged images (ACE+/GFAP+, yellow) demonstrate the colocalization of ACE within reactive astrocytes (indicated by white arrows). Zoom Scale bar = 200 μm. (**B**) Quantification of ACE+/GFAP+ double-positive cells across the experimental groups. (**C**) Pearson’s correlation coefficient analysis, indicating the high degree of colocalization between ACE and GFAP following CFE, which was significantly disrupted by captopril treatment. Data are expressed as mean ± SEM. * *p* < 0.05, ** *p* < 0.01 compared to the Sham group; # *p* < 0.05, compared to the CFE 2 h group.

Given the critical role of inducible nitric oxide synthase (iNOS) in neurotoxicity, we evaluated whether captopril treatment could suppress its expression. FE significantly upregulated both iNOS and IL-1β; these effects were substantially alleviated by captopril (Figure 4). Immunofluorescence staining indicated that CFE-induced ACE overexpression predominantly colocalized with GFAP-positive astrocytes rather than neurons, highlighting a glia-driven neuroinflammatory response (Figure 5).

**Figure 6:**
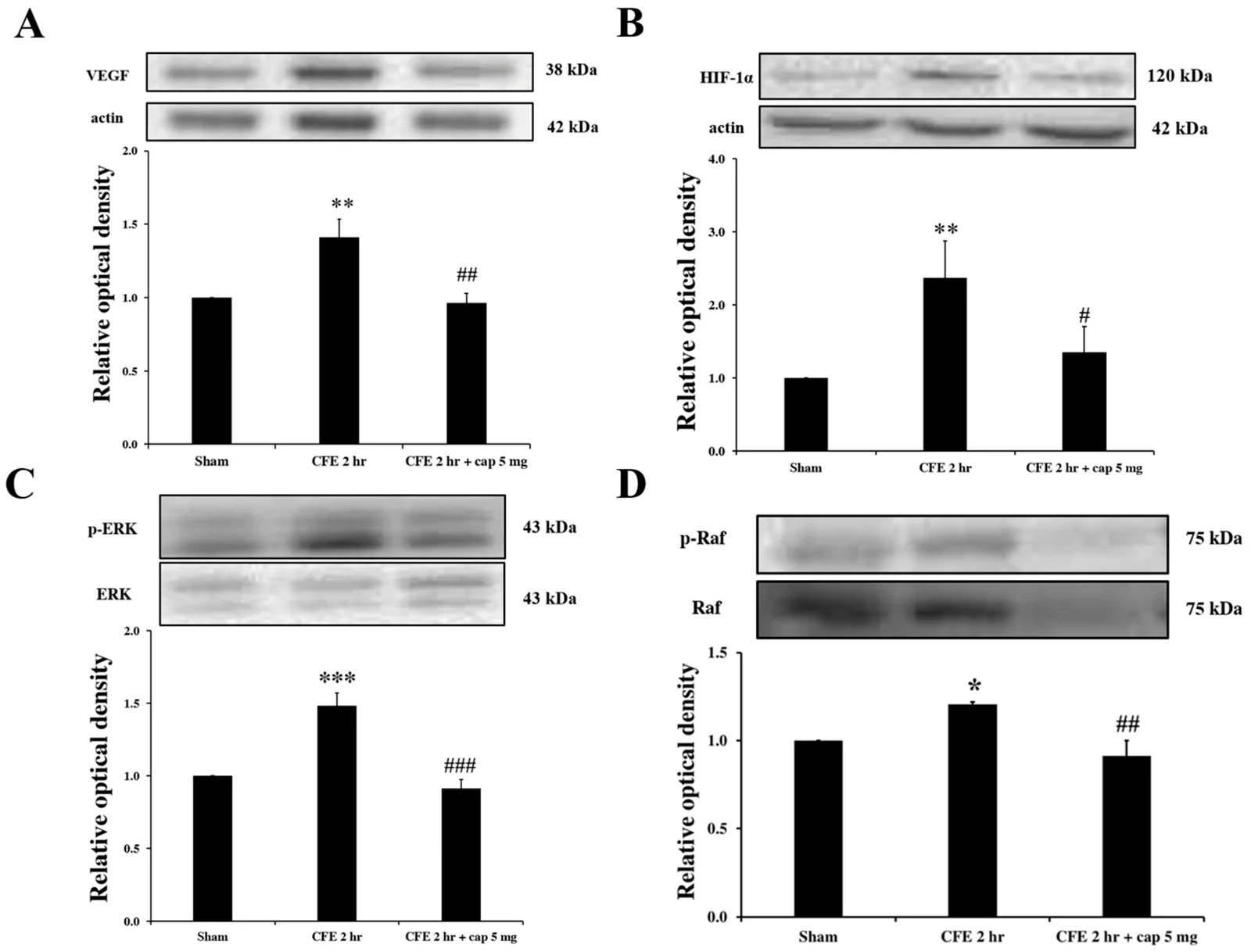
Western blot analyses and quantification of relative optical densities for (**A**) VEGF, (**B**) HIF-1α, (**C**) phosphorylated ERK (p–ERK) relative to total ERK, and (**D**) phosphorylated Raf (p–Raf) relative to total Raf in the cerebral cortex of the Sham, CFE 2 h, and CFE 2 h + captopril (5 mg/kg) groups. β-actin was used as an internal loading control for VEGF and HIF–1α, while total ERK and total Raf served as controls for their respective phosphorylated forms. Data are expressed as mean ± SEM. * *p* < 0.05, ** *p* < 0.01, *** *p* < 0.001 compared to the Sham group; # *p* < 0.05, ## *p* < 0.01, ### *p* < 0.001 compared to the CFE 2 h group.

Because our previous studies demonstrated that FE induces VEGF and HIF–1α expression during pulmonary injury [10,26], we hypothesized that a similar hypoxic and angiogenic response occurs in the cortex. FE significantly upregulated VEGF and HIF–1α expression in cortical tissue, leading to the activation of the ERK signaling cascade. Captopril administration substantially attenuated this CFE-induced upregulation of VEGF (Figure 6A), HIF–1α (Figure 6B), and ERK phosphorylation (Figure 6C).

These findings suggest that the neuroprotective effects of captopril against CFE are associated with the inhibition of the ACE/AngII pathway, which subsequently reduces Ang II release and neuroinflammation. Additionally, captopril upregulates Ang-(1–7) levels, reinforcing its anti-inflammatory and anti-oxidative profile. The attenuation of the VEGF, HIF-1α, and ERK cascade further highlights the complex neuroprotective mechanisms elicited by captopril, providing a strong preclinical foundation for its therapeutic application in managing CFE.

## 4. Discussion

This study demonstrates that FE induces significant acute cerebral damage, characterized by cortical fat deposition, brain edema, elevated lipid peroxidation, and distinct neuronal injury. The resulting pathology is driven by a marked upregulation of cortical ACE, which peaks 2 h post-injury. Administration of the specific ACE inhibitor captopril effectively attenuates this CFE-induced brain edema, oxidative stress, and neuronal damage. The underlying mechanism involves not only the suppression of ACE and its downstream pro-inflammatory effector, Ang II, but also the reduction in key neuroinflammatory mediators such as iNOS and IL–1. This protective effect is accompanied by a restoration of the beneficial peptide Ang-(1–7) and a concurrent mitigation of hypoxia- and inflammation-related pathways, including the VEGF, HIF-1α, and ERK cascades. These combined observations suggest that CFE stems from both the direct neurotoxicity of FFAs and, crucially, the secondary systemic hypoxic and inflammatory cascades originating from the initial pulmonary insult. By modulating the ACE/Ang II axis and its associated neuroinflammatory responses, captopril represents a promising therapeutic approach against this complex CFE-induced cerebral insult.

Neuroinflammation is central to the pathogenesis of CFE, characterized by the chronic release of proinflammatory mediators across various cerebral tissues. While existing literature suggests that during the biochemical phase of CFE, the degradation of fat emboli releases FFAs that induce severe oxidative stress, disrupt the BBB, and activate Toll-like receptor (TLR) signaling in neural cells [21,22,23], our findings, viewed through the lens of the lung-brain axis, indicate that systemic hypoxia originating from the initial pulmonary insult acts as a critical co-conspirator. This combined FFA- and hypoxia-driven inflammatory microenvironment likely serves as a catalyst for RAS dysregulation. In the present study, we found that the RAS plays a critical role in regulating this neuroinflammatory response, promoting an oxidative and proinflammatory environment that accelerates neural damage, consistent with previous reports [29,30]. During CFE and other neurodegenerative conditions, cerebral Ang II levels are significantly upregulated, driving the release of proinflammatory cytokines such as IL-1β, IL-6, and TNF-α, which further exacerbate neuroinflammation and neuronal injury [31,32]. Beyond stimulating cytokine production, Ang II induces COX-2 expression, a critical step for sustained inflammatory responses [30]. The inflammatory effects of Ang II are primarily mediated by the AT1R, which triggers key signaling pathways, including the MAPK cascade and NF-κB. This cascade stimulates proinflammatory mediators and oxidative stress, exacerbating neuronal death in various pathological conditions [10,26,33,34,35]. AT1R activation is a potent driver of oxidative stress; extensive evidence from other neurodegenerative models demonstrates that Ang II accumulation exacerbates dopaminergic neuronal death by amplifying intracellular reactive oxygen species (ROS) production [36]. This aligns with our observation of rapidly elevated cortical MDA levels following FE. By administering captopril, we effectively reduced upstream Ang II production, thereby preventing this AT1R-mediated damage. We also found that captopril not only attenuated ACE expression and downregulated Ang II but also upregulated cerebral Ang-(1–7) levels, further suppressing CFE-induced neuroinflammation. This comprehensive shift in the RAS balance can be explained by the fact that the administration of captopril or candesartan (another angiotensin II receptor blocker, ARB) not only stimulates ACE2 expression but also increases its enzymatic activity [37]. Because ACE2 actively cleaves Ang II to generate Ang-(1–7), this enhanced ACE2 activity perfectly accounts for both the reduction in Ang II and the simultaneous elevation of Ang-(1–7). Furthermore, since captopril is primarily an ACE activity inhibitor and we did not evaluate mRNA levels, the observed reduction in ACE protein expression is likely an indirect consequence of this process. By driving the depletion of Ang II and mitigating the overall inflammatory microenvironment, this ACE2-mediated mechanism disrupts a positive feedback loop that would otherwise maintain high ACE expression. The counter-regulatory ACE2/Ang-(1–7)/MasR axis and the AT2R exert well-documented anti-inflammatory and neuroprotective actions [30,32,38]. Activation of the AT2R and MasR reduces TNF-α levels and increases the release of the anti-inflammatory cytokine IL-10 in both astrocytes and microglia [39]. Ultimately, these receptors mitigate oxidative stress and neuroinflammation by suppressing NADPH oxidase activity and inhibiting key inflammatory pathways such as the MAPK cascade and NF-κB [40].

It is also worth noting that the brain RAS network extends beyond the ACE2 axis. Ang II is actively metabolized into Ang IV, adding another variable to this system. Aminopeptidase A converts Ang II to Ang III, which aminopeptidases N and B then cleave to form Ang IV [41]. The exact function of Ang IV in neuroinflammation remains under debate. Several studies report neuroprotective and antioxidant benefits, including reduced hippocampal oxidative stress following brain injury [42,43]. However, other findings suggest that acute Ang IV exposure might actually trigger NF-κB activation and upregulate inflammatory genes [35,44]. Because our current study focused primarily on the classical and ACE2/Ang-(1–7) pathways, we did not directly measure Ang IV levels. Given its controversial effects, determining whether captopril alters the Ang IV pathway—and how that might contribute to or counteract neuroprotection in CFE—will be an important direction for future studies.

Glial activation is a hallmark of RAS-driven neuroinflammation [42,45]. In the present study, immunofluorescence analysis revealed that CFE-induced ACE upregulation occurred predominantly in GFAP-positive astrocytes rather than neurons. In the context of the lung-brain axis, these reactive astrocytes likely act as the first responders to the systemic hypoxia and inflammatory signals crossing the compromised BBB following the initial pulmonary embolism. As a primary source of local Ang II production under pathological stress, reactive astrocytes can drive a paracrine signaling cascade that activates adjacent microglia [46,47]. Ang II exposure directly triggers microglial activation, leading to the sustained release of cytokines and reactive oxygen species [35]. Recent evidence also indicates that microglia express intrinsic RAS components, including ACE and ACE2, allowing them to actively modulate local neuroinflammatory networks rather than merely responding to astrocyte-derived signals [48]. By inhibiting ACE, captopril appears to interrupt this pathogenic astrocyte-microglia crosstalk, effectively suppressing the broader glia-mediated inflammatory response initially triggered by the pulmonary insult. Beyond driving inflammation, astrocyte dysfunction and elevated Ang II levels also compromise the integrity of the blood–brain barrier (BBB). Ang II facilitates the breakdown of the BBB, promoting the infiltration of peripheral immune cells and circulating pulmonary inflammatory mediators, and the development of vasogenic edema [49,50,51]. This edema subsequently increases intracranial pressure, which further exacerbates neuronal damage. In this study, captopril administration significantly attenuated CFE-induced cerebral edema and neuronal injury, highlighting its potential as a targeted therapeutic strategy for preserving BBB integrity and mitigating brain edema driven by cross-organ inflammatory signaling.

Our results demonstrate that captopril administration effectively suppresses the cortical HIF-1α/VEGF/ERK pathway. HIF-1α is a master transcriptional regulator exquisitely sensitive to hypoxia. In our untreated CFE group, the marked upregulation of cerebral HIF-1α promotes VEGF overexpression, which severely disrupts the blood–brain barrier (BBB), increases vascular permeability, and exacerbates vasogenic brain edema [26,30,34]. Crucially, this hypoxic response tightly intertwines with the RAS to create a self-amplifying vicious cycle of edema and inflammation [49,50]. Specifically, HIF-1α not only upregulates ACE expression [52] but is also suggested to suppress ACE2, driving further Ang II accumulation. Concurrently, VEGF itself can induce ACE through tyrosine kinase, PKC, and cGMP pathways [53]. As Ang II accumulates, its binding to the AT1 receptor triggers oxidative stress and activates MAP kinases, particularly ERK. Together with elevated ROS, this ERK activation stabilizes HIF-1α, which in turn drives continuous VEGF transcription [26,34]. By downregulating the ERK/HIF-1α/VEGF axis, captopril effectively breaks this vicious cycle. This prevention of VEGF overproduction likely explains the reduced brain edema observed in our captopril-treated groups, highlighting captopril’s dual protective mechanism in mitigating both neuroinflammation and vascular damage following pulmonary-induced hypoxia.

The neuroprotective effects of captopril against CFE are primarily driven by ACE inhibition, which effectively blunts Ang II-mediated neuroinflammation. This reduction in Ang II is accompanied by elevated Ang-(1–7) levels, providing an additional layer of antioxidative and anti-inflammatory defense. The observed suppression of the HIF-1α/VEGF/ERK signaling axis further reveals a truly multifaceted neuroprotective profile. These combined insights establish a compelling preclinical foundation for the therapeutic application of captopril in managing CFE, highlighting the critical importance of targeting the lung-brain axis to preserve neuronal health following severe pulmonary insults.

## 5. Conclusions

Cerebral fat embolism is a consequence which is driven by hypoxia and RAS overactivation. By inhibiting ACE, captopril effectively modulates this axis, reducing oxidative stress and neuroinflammation. Targeting the ACE/Ang II pathway represents a promising therapeutic strategy for managing the neurological complications of FE syndrome.

## Data Availability

All data generated or analyzed during this study are included in this published article.
